# The Relationship Between e-Health Literacy and Breast Cancer Literacy Among Saudi Women

**DOI:** 10.3389/fpubh.2022.841102

**Published:** 2022-04-06

**Authors:** Alia Almoajel, Samar Alshamrani, Mesnad Alyabsi

**Affiliations:** ^1^Department of Community Health Sciences, College of Applied Medical Sciences, King Saud University, Riyadh, Saudi Arabia; ^2^Population Health Research Section, King Abdullah International Medical Research Center, Riyadh, Saudi Arabia; ^3^King Saud bin Abdulaziz University for Health Sciences, Riyadh, Saudi Arabia

**Keywords:** breast cancer literacy, e-health literacy, predictor, Saudi women, digital health (e-health)

## Abstract

Breast cancer is the most common cancer in women and represents a significant burden among women worldwide. The concept of health literacy is relatively new to the Gulf states, particularly to Saudi Arabia. Research on this topic is scarce, and no study has empirically explored the influence of e-health literacy on breast cancer literacy. The purpose of this study was to measure the impact of e-health literacy on breast cancer literacy among Saudi women in Riyadh city, Saudi Arabia. A cross-sectional survey was conducted online in a cohort of 336 women and disseminated via social media using the e-Health Literacy Scale (eHEALS) and Breast Cancer Literacy Assessment Tool (Breast-CLAT). Multiple regression analysis was executed to identify the sociodemographic factors that influence the e-health literacy and breast cancer literacy of participants. The participants showed high level of e-health literacy with total eHEALS score of 28.79, and better breast cancer literacy with total Breast-CLAT score of 23.44. This study yielded three significant findings: (1) e-health literacy is influenced by age and education which implies that youngest participants showed higher eHEALS scores than their older counterparts and that having increased education level reflected increased eHEALS scores, (2) breast cancer literacy is predicted by education and income which suggests that those who have higher levels of education and higher monthly incomes have increased levels of breast cancer literacy, and (3) breast cancer literacy and e-health literacy were associated which shows that participants with higher eHEALS scores were more likely have increased level of breast cancer literacy. The study revealed that the participants had high level of e-health literacy which correlated to their increased level of breast cancer literacy. The study findings implied that it is important for Saudi women to possess high levels of e-health literacy about breast cancer as more breast cancer educational resources are online nowadays.

## Introduction

Breast cancer is the most common cancer among women, and its magnitude and incidence are rising worldwide (1). Globally, there are 1.7 million cases of breast cancer diagnosed annually, and its prevalence among Saudi women is about 21.8% (2), whereas it is only 14% among American women (3). Timely screening and early detection for breast cancer are associated with reduced morbidity and mortality (4). Likewise, many factors contribute to the decision to undergo breast cancer screening, including age, marital status, education, socioeconomic status, usual source of care, doctor's recommendation, access to healthcare, and social support. Another important factor of cancer screening is e-health literacy (5).

According to Zakaria et al. (5), e-health literacy is the ability to search for, find, comprehend, and appraise health information from electronic sources, and apply the knowledge gained to address or solve a health problem. The concept of health literacy is relatively new to the Gulf states, particularly to Saudi Arabia, and research on this topic is scarce (6). On the other hand, breast cancer literacy includes the awareness of cancer patients of the resources for screening and knowledge, as well as prevention and control of breast cancer (7). Accordingly, women must acquire the skills to identify, use, and effectively engage with different health resources on the Internet concerning breast cancer. Moreover, evidence shows that the e-health strategy has the potential to address the needs of a growing number of breast cancer patients (8). Breast cancer patients require sufficient e-health literacy in order to use Internet health recourses more effectively to improve their health outcomes (7). However, there is little evidence on how e-health literacy is directly related to breast cancer literacy. Despite the many health resources available online, many breast cancer patients may have difficulty finding useful information about screening or prevention of breast cancer due to their low level of e-health literacy.

Evidence has shown that in the US (9), 79% of patients diagnosed with breast cancer demonstrated high health literacy, while 21% demonstrated low health literacy, and were less likely to access the Internet for healthcare information. The same study revealed that low health literacy was associated with a high school level education (9). In another recent study conducted in the US (10), women with breast cancer with lower health literacy demonstrated the strongest correlation between their cancer care coordination and quality of life. A third US study evaluated the applicability of a multidimensional framework to explore factors (e.g., cultural attitudes, beliefs and practices, and facilitators of and barriers to cancer literacy) and found that the framework increased the understanding of those factors that influence an individuals' approach to cancer prevention and screening (11). Moreover, Sinicrope et al. (12) developed a home-based breast cancer mammography intervention strategy among US Navajo women, highlighting the necessity of tailoring educational materials to the participants' culture and language, and emphasizing the effects of a support system.

In Australia, the application of an e-health platform called “Healthy.me” was reported to be a useful online resource for breast cancer patients. This platform addresses the needs of a growing population of breast cancer survivors, as well as the integration of healthcare needs of women with breast cancer (8). According to Zhou and Wang (13), Chinese cancer survivors frequently must acquire adequate e-health literacy and active social support from online health communities in order to benefit from using online resources to improve their health. In France, Ousseine et al. (14) reported that cancer survivors who had limited health literacy were more frequently noted to avail follow-up visits with general practitioners and social workers, which required these healthcare workers to undergo additional training to effectively communicate with and support these vulnerable patients.

In a study in Saudi Arabia, Binhussien and Ghoraba (15) found that books, printed material, and the Internet were most commonly cited by participants as source of information of breast cancer while social media, in particular, represented the largest source of information. Another previous Saudi study conducted in Al-Qassim region explored the knowledge, attitudes, and practices surrounding breast cancer and screening in Saudi female teachers. The study reported insufficient knowledge in female teachers of breast cancer (16). Similarly, the findings of Al Otaibi et al. (17) and Alsolami et al. (18) found that the knowledge, awareness, and practices of Saudi females regarding breast cancer are relatively poor, and requires increased societal attention. Moreover, Zakaria et al. (5) argued that apart from research noting the lack of available metrics, no major studies have been conducted measuring e-health literacy at the population level in Saudi Arabia. Thus, it is crucial to study e-health literacy among Saudi women diagnosed with breast cancer to measure and maximize the potential impact of Internet resources on their health. This study explored how e-health literacy is associated with breast cancer literacy in Saudi women, using the sociodemographic characteristics of the participants as predictor variables. The purpose of this study was to empirically explore the relationship between e-health literacy and breast cancer literacy among Saudi women.

## Materials and Methods

### Research Design

This quantitative study used a correlational, cross-sectional design. This study adhered to the Strengthening the reporting of observational studies in epidemiology (STROBE) guidelines for cross-sectional studies.

### Sample and Setting

This study was performed using answers collected from random sample of 336 Saudi women in Riyadh, Saudi Arabia. Using the G^*^Power version 3.1.7 software as the basis for the sample size computation, the total sample of 326 participants was sufficient to yield a large effect size (0.80) at 5% margin of error and 95% confidence level. The following were the criteria for inclusion in this study: women must (1) be Saudi citizens, (2) be 18 years old or above, (3) live in Riyadh as the capital city of the country, and (4) give consent to participate. Saudi minors and non-Saudi women were excluded from participating in this study.

### Instrumentation

We built an online questionnaire for this study according to literatures and it consisted of three parts. The first part included demographic variables such as age, gender, marital status, and number of children. Additionally, socioeconomic variables such as education, employment status, monthly income, and health insurance status were included. The second part utilized the e-health literacy scale (eHEALS), which determined the participants' combined knowledge, confidence, and perceived skills at finding, evaluating, and applying electronic health information to health problems (19). The scale consisted of 8 items scored on a 5-point Likert scale: strongly disagree (1) to strongly agree (5) like “I know what health resources are available on the Internet,” Strongly Disagree (1), Disagree (2), Undecided (3), Agree (4), and Strongly Agree (5). The score of each item was calculated to obtain the mean of all Items. The internal consistency of the data collected using the eHEALS in this study was high, with a Cronbach alpha score of 0.90. The third part of the questionnaire measured the breast cancer literacy of the participants using the Breast Cancer Literacy Assessment Tool (Breast-CLAT) (7). The Breast-CLAT is a 34-item assessment that measures breast cancer literacy in three domains: (a) awareness (items 1–6), (b) knowledge and screening (items 7–19), and (c) prevention and control (items 20–34). This instrument used a multiple choice and true/false format, and was scored as a binary variable (0 = incorrect, 1 = correct), like “A mammogram can cause breast cancer to spread?*"*, True (1) and False (0). The mean of each dimension was calculated to obtain the dimension score. The internal consistency of the data collected using the Breast-CLAT in this study was high, with a Cronbach alpha score of 0.73.

### Ethical Consideration and Data Collection

The study protocol was approved by the Institutional Review Board (IRB) at King Abdullah International Medical Research Center in Saudi Arabia (Ethical Approval Number: IRB#139-RC20/142/R). Furthermore, permission from the copyright holders of the tools was obtained before data gathering. The online surveys were disseminated in English language with Arabic translation via social media such as Facebook, Snapchat, Twitter and WhatsApp which are commonly used in Saudi Arabia. Throughout the study, the researchers adhered with the guidelines and ethical standards in conducting research studies in human participants required by the IRB and the Declaration of Helsinki and its revisions. A research information sheet containing a brief background, importance, and objectives of the study, as well as the necessary voluntary participation and rights of Saudi women information was included in the introductory section of the online survey. Saudi women who clicked “Yes, I agree” to the online consent were directed to continue with the survey and those who refused to participate were instructed to leave the survey. No identification information was collected from the participants to ensure privacy and confidentiality throughout the study. Additionally, no incentives were given for participation. Data was protected by password, and only the authors had access to the online documents.

### Statistical Analysis

Data processing and analyses were carried out using the statistical software IBM SPSS for Windows version 26. To determine Saudi women's e-health and breast cancer literacy, descriptive statistics such as frequency and percentages were used in addition to means and medians. Multivariate linear regression was used to determine whether demographic variables could predict overall eHEALS and Breast-CLAT scores. Finally, a Pearson correlation analysis was conducted to determine the relationship between e-health literacy and breast cancer literacy. Analyses were considered statistically significant at *p* < 0.05.

## Results

### Sociodemographic Characteristics

The sociodemographic characteristics of 336 Saudi women participants, shown in Table 1, indicate an age range from 18 to 47 years old (mean = 24 years old, SD = 1.48 years). The majority of the respondents were unmarried with no children, had earned a college or associates degree, and did not have health insurance. The highest proportion of the respondents were students; from the participants who were employed, the highest portion reported a monthly income that ranged from 10,000 to 20,000 Saudi riyals (2,665–5,330 US dollars).

**Table 1:** Sociodemographic characteristics of Saudi women (*N* = 336).

**Sociodemographic characteristics**		***N***	**%**
Age (in years)	18–24	145	43.2
	25–32	50	14.9
	33–39	43	12.8
	40–46	56	16.7
	47 and above	42	12.5
Marital status	Married	152	45.2
	Unmarried	184	54.8
Number of children	No children	180	53.6
	1–3 children	63	18.8
	4 and more children	93	27.7
Education	High school level or less	54	16.1
	College or associate degree	262	78.0
	Postgraduate degree	20	6.0
Employment	Student	131	39.0
	Employed	113	33.6
	Unemployed	80	23.8
	Retired	12	3.6
Income (SAR)	More than 20,000 (More than 5,330 USD)	73	21.7
	10,000–20,000 (2,665–5,330 USD)	134	39.9
	5,000–10,000 (1,333–2,665 USD)	85	25.3
	Less than 5,000 (Less than 1,333 USD)	44	13.1
Health insurance	No	219	65.2
	Yes	117	34.8

### e-Health Literacy of Saudi Women

The e-health literacy total score of the respondents on the eHEALS was 28.79 (SD = 6.75) (Table 2). Most respondents agreed with the following seven statements on the eHEALS: “I know how to use the Internet to answer my health questions” (69.9%), “I know how to use the health information I find on the Internet to help me” (68.7%), “I know how to find helpful resources on the Internet” (63.6%), “I know where to find helpful health resources on the Internet” (61.0%), “I know what health resources are available on the Internet” (59.3%), “I have the skills I need to evaluate the health resources I find on the Internet” (55.4%), and “I can tell high quality from low quality health resources on the Internet” (53.6%). The only statement with which fewer than half of the women agreed (39.6%) was “I feel confident in using information from the internet to make health decisions.”

**Table 2:** e-Health literacy of Saudi women (*N* = 336).

**Items**	**Disagree**		**Undecided**		**Agree**	
	***N***	**%**	***N***	**%**	***N***	**%**
Q1: I know how to find helpful health resources on the Internet.	40	11.9	91	27.1	205	61.0
Q2: I know how to use the Internet to answer my health questions.	29	8.7	72	21.4	235	69.9
Q3: I know what health resources are available on the Internet.	43	13.1	92	27.7	201	59.3
Q4: I know where to find helpful health resources on the Internet.	37	11.0	85	25.3	214	63.6
Q5: I know how to use the health information I find on the Internet to help me.	29	8.7	76	22.6	231	68.7
Q6: I have the skills I need to evaluate the health resources I find on the Internet.	46	13.4	105	31.3	185	55.4
Q7: I can tell high quality from low quality health resources on the Internet.	53	15.8	103	30.7	180	53.6
Q8: I feel confident in using information from the Internet to make health decisions.	84	25.0	119	35.4	133	39.6
**e-Health literacy** [total score (SD) = 28.79 (6.75)]						

### Breast Cancer Literacy of Saudi Women

The total score of the breast cancer literacy of the participants was 23.44 (SD = 3.56) (Table 3). In the breast cancer awareness section, over 80% of women answered the following questions correctly: “If someone hits my breast, I will get breast cancer” (92.6%), “Breastfeeding a baby can protect you from getting breast cancer” (85.7%), and “Women who have large breasts are more likely to get breast cancer than women who have small breasts” (85.4%). However, 58.6% of the respondents were incorrect in responding to the statement: “Although there are many different names for types of cancers you can get them the same way.” Meanwhile, in the knowledge and screening section, over 90% of women answered the following questions correctly: “A mammogram should be done” (93.5%) and “A mammogram can cause breast cancer to spread” (90.5%), but the majority of women (68.2%) were incorrect in answering the question: “Which of these commonly used screening practices are the same?.”

**Table 3:** Breast cancer literacy of Saudi women (*N* = 336).

**Items**	**Correct**		**Incorrect**	
	***N***	**%**	***N***	**%**
**Awareness [mean score (SD) = 4.28 (1.07)]**				
1. Although there are many different names for types of cancers you can get them the same way.	139	41.4	197	58.6
2. Breast cancer is disease.	227	67.6	109	32.4
3. If someone hits my breast I will get breast cancer.	311	92.6	25	7.4
4. The following can lead to getting breast cancer.	185	55.1	151	44.9
5. Women who have large breasts are more likely to get breast cancer than women who have small breasts.	287	85.4	49	14.6
6. Breastfeeding a baby can protect you from getting breast cancer	288	85.7	48	14.3
**Knowledge and screening [mean score (SD)** **=** **9.32 (2.32)]**				
7. Who does a breast-self-examination?	272	81.0	64	19.0
8. A breast-self exam should be done.	252	75.0	84	25.0
9. When doing a breast-self exam I should use.	211	62.8	125	37.2
10. Swelling of all or part of a breast (even if no lump is felt) is a possible sign of breast cancer.	208	61.9	128	38.1
11. Who does a clinical breast examination?	245	72.9	91	27.1
12. A clinical breast examination should be done.	285	84.8	51	15.2
13. Who does a mammogram?	272	81.0	64	19.0
14. A mammogram should be done.	314	93.5	22	6.5
15. Which of these commonly used screening practices are the same?	107	31.8	229	68.2
16. Which of these statements are true?	189	56.3	147	43.8
17. A mammogram can cause breast cancer to spread.	304	90.5	32	9.5
18. You only need to get a mammogram if you have been diagnosed with breast cancer.	200	59.5	136	40.5
19. Getting a yearly mammogram beginning at age 40 decreases my chances of dying from breast cancer.	272	81.0	64	19.0
**Prevention and control** [mean score (SD) = 9.85 (1.73)]				
20. Getting yearly screenings increases a woman's chance of surviving from breast cancer.	326	97.0	10	3.0
21. Getting breast cancer screenings does not mean I will not get breast cancer.	309	92.0	27	8.0
22. If I find a lump under my arm, I should.	264	78.6	72	21.4
23. My family's breast cancer history or prostate cancer history might mean I should begin getting a mammogram earlier than age 40.	312	92.9	24	7.1
24. If one family member has breast cancer, I am at higher risk for getting it.	236	70.2	100	29.8
25. I am at risk for getting breast cancer.	173	51.5	163	48.5
26. Women who are severely overweight increase their risk of getting breast cancer.	167	49.7	169	50.3
27. Women who eat a lot of high fat foods increase their risk of getting breast cancer.	189	56.3	147	43.8
28. I am confident that I know how to do my own breast-self-examination correctly.	144	42.9	192	57.1
29. I can reduce my chances of getting breast cancer by.	295	87.8	41	12.2
30. I can prevent breast cancer by taking vitamins.	232	69.0	104	31.0
31. Resources for breast cancer screening are available for women without health insurance.	294	87.5	42	12.5
32. Do you know where you would get the breast cancer screening for women with health insurance?	158	47.0	178	53.0
33. There are programs for breast cancer screening for a small fee.	67	19.9	269	80.1
34. I know how to help my family member get in a low cost breast cancer screening program.	143	42.6	193	57.4
**Breast cancer literacy** [total score (SD) = 23.44 (3.56)]				

Finally, in the control and prevention section, over 90% of women answered the following questions correctly: “Getting yearly screenings increases a woman's chance of surviving from breast cancer” (97%), “My family's breast cancer history or prostate cancer history might mean I should begin getting a mammogram earlier than age 40” (92.9%), and “Getting breast cancer screenings does not mean I will not get breast cancer” (92%). However, more than 50% of the respondents were incorrect in answering the following questions: “Women who are severely overweight increase their risk of getting breast cancer” (50.3%), “I am confident that I know how to do my own breast-self-examination correctly” (57.1%), “Do you know where you would get the breast cancer screening for women with health insurance?” (53.0%), “I know how to help my family member get in a low cost breast cancer screening program” (57.4%), and “There are programs for breast cancer screening for a small fee” (80.1%).

### Relationship Between e-Health Literacy and Breast Cancer Literacy of Saudi Women

E-health literacy was moderately correlated with breast cancer literacy in the participants of this study (*r* = 0.30; *p* < 0.001). The overall multiple regression model accounted for 40% of the variance in eHEALS scores, which was statistically significant (*R*^2^ = 0.62; $R_{\text{adjusted}}^{2}$ = 0.59; *F* = 2.97; *P* < 0.05). The summary of the regression coefficients generated by this analysis is shown in Table 4. Statistically significant predictors of e-health literacy included age and education. Total eHEALS score decreased by 0.26 points for each year of age increased (β = −0.26; *p* = 0.02). This indicates that, on average, the youngest participants (18 years old) were likely to score higher on the eHEALS than older adults. In addition, as education level (β = 0.11; *p* = 0.04) increased, total eHEALS scores also increased. Marital status, number of children, employment status, income, and health insurance status were not significantly associated with e-health literacy.

**Table 4:** Multiple linear regression for e-Health literacy and breast cancer literacy (*N* = 336).

**Predictor variables**	**Unstandardized coefficients**		**Standardized coefficients**	***P*-value**
	**B**	**Standard error**	**β**	
**e-Health literacy**				
Age	−1.171	0.501	−0.26	0.02^*^
Marital status	−1.782	1.287	−0.13	0.17
Number of children	1.189	0.944	0.16	0.21
Education	1.613	0.799	0.11	0.04^*^
Employment	0.337	0.572	0.05	0.56
Income	−0.023	0.390	0.003	0.95
Health insurance	1.379	0.752	0.01	0.68
**Breast cancer literacy**				
Age	0.147	0.258	0.06	0.57
Marital status	−0.422	0.662	−0.06	0.52
Number of children	−0.608	0.485	0.15	0.21
Education	1.194	0.411	0.16	0.004^*^
Employment	−0.125	0.294	0.03	0.67
Income	0.700	0.201	0.19	0.001^**^
Health insurance	0.250	0.388	0.03	0.52

*e-Health literacy and breast cancer literacy were dependent variables.*

*R^2^ = 0.62; adjusted R^2^ = 0.59.*

* *p < 0.05.*

** *p < 0.01*.

Meanwhile, multivariate analyses of breast cancer literacy showed that women who had higher levels of education showed increased levels of breast cancer literacy (β = 0.16; *p* = 0.004), and those who have higher monthly incomes also have increased levels of breast cancer literacy (β = 0.19; *p* = 0.001). Age, marital status, number of children, employment status, and health insurance status were not significantly associated with breast cancer literacy.

## Discussion

The findings of this study provide insights about the e-health and breast cancer literacy among women in Riyadh City, Saudi Arabia. To our knowledge, this is the first study to empirically measure the influence of e-health literacy on breast cancer literacy among Saudi women. The findings of this study argue that by understanding e-health literacy levels, healthcare providers will be able to create a cancer patient-education system to increase the awareness, knowledge of screening methods, and the preventive and control measures for breast cancer. This study yielded three significant findings: (1) the factors that influence e-health literacy are age and education, (2) the factors that influence breast cancer literacy are education and income, and (3) there are relationships between breast cancer literacy and e-health literacy.

These results implied that younger Saudi women showed higher e-health literacy compared to their older counterparts. This finding is consistent with that of Hoogland et al. (20), where e-health literacy average scores were significantly higher in younger cancer participants than older ones. Correspondingly, this study indicated that respondents with higher educational levels displayed higher e-health and breast cancer literacy. Moreover, participants with higher income reported higher breast cancer literacy. A previous study in Saudi Arabia argued that low income is one of the major impediments in screening for early detection and prevention of breast cancer (18), although this was not found to be the case as per the study participants in the Riyadh region. However, only 23.8% of the respondents in this study were unemployed, unlike with the study of Alsolami et al. (18) in which most of the women in Makkah region self-reported as unemployed. However, the findings of this study contradict those of a previous US study that found socioeconomically disadvantaged cancer patients, who in particular reported having lower income and education, were at risk for not having follow-up care discussions with their healthcare providers (21). On the other hand, a recent study in the United States revealed that age and education did not correlate with the usage of the internet to seek healthcare information among breast cancer patients in South Texas. Moreover, level of education was also not associated with the participants' awareness about breast cancer and reconstructive options (9).

In this study, higher education and income levels were found to be significantly associated with high breast cancer literacy. Although education, age, and language were found to be critical factors among research participants in the study of Arshad et al. (22), there still are numerous other dynamics, such as culture and religion, that influence these women's e-health and breast cancer literacy that were not explicitly captured in this study. As this study did not measure such factors, the findings are restricted to revealing significant influence of demographic factors such as age, education, and income. However, a recent study showed that studying cancer literacy in a multidimensional framework increased the influence of factors directed to an individuals' approach to cancer screening and prevention (11). This framework included factors other than sociodemographic characteristics, such as attitudes, barriers, beliefs, facilitators, and practices among diverse populations of white, African-American, and Latino individuals. However, according to Echeverri et al. (11), barriers to survivorship care, which were strongest among breast cancer patients with low health literacy, included low level of education, residing in rural areas, and socioeconomic status. Moreover, another recent US study explored other influencing variables, and indicated a strong correlation between coordination cancer care and quality of life among breast cancer survivors who have lower health literacy (10).

In this study, slightly over half of the respondents demonstrated a high e-health literacy level. E-health literacy, including knowledge, skills, and confidence, increase with level of education, perhaps due to the greater use of the internet and the use of social media. Conversely, e-health literacy decreases with age. Overall, participants in this study lacked confidence in their ability to use the information found on the internet to make health decisions, and their ability to distinguish between high- and low quality health resources on the internet. This finding was supported by another previous study, which reported that the respondents felt less confident in their ability to evaluate web-based health information (23). Aside from web-based health information, there are other innovative strategies that can enhance e-health literacy for cancer patients such as the use of standardized Teach-Back method to check the understanding and engagement of patients (24), the Cancer 101 digital videos that described cancer in simple language (25), and mobile applications, telehealth and wearable devices (26). A separate study noted that educational background influenced e-health literacy in the general population (27). Another previous study demonstrated an association between health literacy and breast cancer knowledge (28), which indicates that participants with limited health literacy have less breast cancer knowledge, and less perceived severity than those who had higher health literacy scores.

Furthermore, these results also indicated that e-health literacy was moderately correlated with breast cancer literacy. This means that initiatives to improve e-health literacy skills and knowledge may also likely increase the awareness, knowledge of screening modalities, and prevention and control of breast cancer. This might be helpful to increase the effectiveness of breast cancer education, improve the management of cancer diagnoses, and increase the rate of screening and preventive measures of breast cancer. Based on this finding, we argue that e-health literacy can influence the awareness, knowledge of screening modalities, and the prevention and control of breast cancer. This argument is consistent with recent evidence that suggests that those who are ignorant of such practices due to being uneducated and unable to use internet resources for breast cancer awareness have greater risks of breast cancer (18). Comparable findings in another previous study found that Iranian women with low health literacy had less knowledge about and perceived severity of breast cancer than those who had higher health literacy scores. However, the results also suggested that the respondents with higher health literacy scores adhered to preventive breast cancer measures, such as performing breast-self-exams more frequently than other Iranian women (28).

## Limitations

This study has some limitations to consider. All the data reflects the participants' self-reported perceptions on their e-health literacy and breast cancer literacy; thus, they might not reflect participants' actual e-health literacy and breast cancer literacy. Furthermore, online surveys make it relatively easy to assess knowledge of levels of e-health literary, but there are limitations when it comes to participants' ability to appraise whether they have the information they require and whether they are appropriate. Also, the online administration of the study hindered the assessment of true functional breast cancer literacy of the participants. Another limitation is that we did not investigate the frequency of the use of the internet, knowledge about breast cancer, and other sources of breast cancer information; therefore, predictors of e-health literacy were limited to demographic factors.

## Conclusions and Relevance for Practice

In conclusion, the study found that the high level of e-health literacy of Saudi women was correlated to their increased level of breast cancer literacy. In addition, the e-health literacy of Saudi women was best predicted by age and education level while education and income levels best predicted their breast cancer literacy.

Identifying women who are at risk for breast cancer is a top concern for any healthcare facility. As more breast cancer educational resources are online nowadays, it is important that women in at home possess high levels of e-health literacy about breast cancer. In this study, the use of health resources on the internet shows positive influence on the breast cancer literacy among women. Assessment of women's e-health literacy will help increase the effectiveness of breast cancer education, screening, and prevention and control programs. This study provides valuable findings that can be used to improve breast cancer literacy among women. These results may become the basis for formulating healthcare policies geared toward consistent and easy access to the internet, which is an immediate source of appropriate health information to women concerning the awareness, knowledge of screening modalities, and prevention and control of breast cancer. Public health workers can use outreach measures for women with lower e-health literacy and breast cancer literacy by illustrating the differences between verified health resources on the internet and those sources with questionable health information.

## Data Availability Statement

The raw data supporting the conclusions of this article will be made available by the authors, without undue reservation.

## Author Contributions

AA, MA, and SA: conceptualization, project administration, validation, writing—original draft, and writing—review and editing. AA: data curation and supervision. MA: formal analysis. SA: investigation. AA and MA: methodology. All authors have read and agreed to the published version of the manuscript.

## Conflict of Interest

The authors declare that the research was conducted in the absence of any commercial or financial relationships that could be construed as a potential conflict of interest.

## Publisher's Note

All claims expressed in this article are solely those of the authors and do not necessarily represent those of their affiliated organizations, or those of the publisher, the editors and the reviewers. Any product that may be evaluated in this article, or claim that may be made by its manufacturer, is not guaranteed or endorsed by the publisher.
